# Human leukocyte antigen (HLA) class I frequencies in human T-cell lymphotropic virus type 1 (HTLV-1)-infected patients from Salvador-Brazil

**DOI:** 10.1186/1742-4690-8-S1-A121

**Published:** 2011-06-06

**Authors:** Viviana N Olavarria, Maria F Grassi, Ramon Kruschewsky, Xiao-Jiang Gao, Alline Nascimento, David Watkins, Mary Carrigton, Bernardo Galvao-Castro

**Affiliations:** 1 Bahiana School of Medicine and Public Health (EBMSP) Salvador, Bahia, Brazil; 2 Advanced Laboratory of Public Health, Gonçalo Moniz Center, Fundação Oswaldo Cruz, Salvador, Bahia, Brazil; 3 Laboratory of Genomic Diversity, SAIC-Frederick, Inc., National Cancer Institute, Frederick, Maryland, 21702, USA; 4 Wisconsin National Primate Center, University of Wisconsin–Madison, Madison, Wisconsin, USA

## Introduction

The development of human T- cell lymphotropic virus type 1 (HTLV-1)-associated myelopathy/tropical spastic paraparesis (HAM/TSP) may be related to genetic factors related to the presentation of viral antigens by human leukocyte antigen (HLA).

## Aim

To determine the HLA class I genotype of HTLV-1-infected patients.

## Methods

420 HTLV-1-infected patients (280 healthy carriers (HC) and 140 HAM/TSP) from the HTLV Reference Center in Salvador, Brazil were evaluated. HLA genotype was performed using automated DNA sequencers and analyzed using the software program Assign-SBT TM 3.2.

## Results

The HLA types most frequently observed in all individuals combined were A*02 (26.1%), A*03 (10.5%); B*35 (13.1%), B*44 (10.4%), Cw*04 (21.9) and Cw* 07 (17.7%). The presence of HLA-A*02 reduced the odds of HAM-TSP (Odds Ratio: 0.4, p<0.0001 and IC95%: 0.28-0.59). The frequency of homozygosity of HLA-A was 8%, 4% for HLA-B and 9% for HLA-C overall. Proviral load was significantly lower in HC among those heterozygous at all three HLA class I loci group relative to HC who were homozygous at one or more HLA class I loci group (p = 0.029). However, no difference in homozygosity between proviral load in the HAM/TSP patients was observed (p = 0.57).

## Conclusion

HLA-A*02 allele is more prevalent in the HC patients, which suggests a protective role of this allele against HAM/TSP. Furthermore, HC patients had a higher frequency of heterozygosity at all three HLA class than that in HAM/TSP patients.

